# Evaluation of cervical vestibular miogenic evoked potential and electrococleography in the diagnosis of vestibular migraine

**DOI:** 10.1016/j.bjorl.2024.101489

**Published:** 2024-09-03

**Authors:** Talita Parente Rodrigues, Viviane Carvalho da Silva, Ana Maria Almeida de Sousa, Tino Miro Aurélio Marques, Emanuel Saraiva Carvalho Feitosa, Marcos Rabelo de Freitas

**Affiliations:** a Universidade de Fortaleza (UNIFOR), Fortaleza, CE, Brazil; b Hospital Universitário Walter Cantídio, Fortaleza, CE, Brazil; c Faculdade de Medicina da Universidade Federal do Ceará (UFC), Fortaleza, Ceará, Brazil; d Departamento de Otorrinolaringologia, Hospital Universitário Walter Cantídio, Fortaleza, CE, Brazil; e Clínica Otorhinos, Fortaleza, CE, Brazil

**Keywords:** Migraine, Vertigo, Evoked potential, Electrocochleography

## Abstract

**Objectives:**

Vestibular migraine is a neurological disorder characterized by the association of vertigo and headache, affecting up to 1% of the population. Among its differential diagnoses is endolymphatic hydrops. The aim of this study was to investigate the role of cervical vestibular-evoked myogenic potential and electrocochleography in the diagnosis of vestibular migraine.

**Method:**

Thirteen women with clinical diagnosis of vestibular migraine (mean age 44 years) and 13 healthy volunteers without auditory and/or vestibular complaints matched for sex and age were evaluated by performing hydrops examinations of cervical vestibular-evoked myogenic potential and electrocochleography.

**Results:**

The presence of vertigo and headache was reported by all members of the group with vestibular migraine, associated with symptoms such as nausea, photophobia, and phonophobia. Tinnitus was the most frequent auditory complaint. A significant increase in P1 and N1 latencies was observed in the test group. There was no significant difference in the occurrence of asymmetry and decreased amplitude of the cervical vestibular-evoked myogenic potential. Electrocochleography showed an increase in amplitude of the summation potential. The altered SP/AP ratio was double in the group with vestibular migration, without statistical significance.

**Conclusions:**

Changes in latency increase of cervical vestibular-evoked myogenic potential suggests a central lesion. Patients with vestibular migraine may present electrocochleography compatible with endolymphatic hydrops.

**Level of evidence:**

Level 4.

## Highlights

• Vestibular Migraine (VM) is a neurological disorder which associates vertigo and headache.
• Ménière's Disease (MD) is the main differential diagnosis of VM.
• cVEMP, caloric test and ECoG suggests VM acts on several pathways in the vestibular system.
• VM may present ECoG compatible with endolymphatic hydrops suggesting MD association.
• Specific markers for the diagnosis of VM were not identified in this study.

## Introduction

Vestibular Migraine (VM) is defined as an incapacitating neurological disorder characterized by vestibular symptoms such as vertigo, dizziness, or imbalance associated with headache. However, headache does not necessarily occur simultaneously with symptoms of vertigo.^1^

In 2012, the Bárány Society published a consensus establishing diagnostic criteria for VM that includes definitive and probable Vestibular Migraine (pMV).^2^

Ménière's Disease (MD) is the main differential diagnosis. Studies have shown a higher prevalence of VM in patients with MD. About 30% of patients with Ménière's disease may have VM.^3^ Currently, there is no definitive diagnostic test that can safely distinguish the two conditions.^4^

The literature suggests the application of vestibular function tests to characterize possible VM anomalies and distinguish VM from MD, particularly cervical Vestibular-Evoked Myogenic Potential (cVEMP), by evaluating the sacculocolic reflex. Significant asymmetries were observed in patients with VM vis-à-vis normal individuals, suggesting saccular dysfunction in VM.^5^, ^6^, ^7^

Electrocochleography (ECoG) is a short latency auditory-evoked potential, which allows recording of bioelectrical events of the cochlea and cochlear nerve resulting from sound stimulation, through analysis of the recording of three phenomena: Cochlear Microphonism (CM), Summation Potential (SP), and Action Potential (AP). The evaluation of cochlear functioning has focused on the amplitude relationship between the SP and AP (SP/AP).^8^

Considering the lack of definitive objective tests to aid in the diagnosis of VM, the objective of this study was to investigate the role of cVEMP and ECoG in the clinical diagnosis of this disease.

## Methods

This study was approved by the Research Ethics Committee of Hospital Universitário Walter Cantídio. All participants signed an Informed Consent Form – ICF.

### Type of study

This was an analytical, descriptive, retrospective case-control study conducted during 11 months.

### Sample

Twenty-nine participants were referred to the Otoneurology Outpatient Clinic of Hospital Universitário Walter Cantídio. For inclusion in this study, criteria of the Bárány Society (2012) were adopted. Exclusion criteria were impossibility of cervical rotation, and external and/or middle ear disease, as assessed by previous otoscopy and medical records.

Of the 29 participants diagnosed with VM, one was male, and 28 were female. One participant was excluded due to the presence of a middle ear disease; 13 did not attend the outpatient clinic for examinations on the previously scheduled date; in two cases (one male and one female), it was not possible to contact the recorded telephone number. Thus, the final sample consisted of 26 participants who were divided equally into two groups: Test Group (TG) and Control Group (CG). In the TG, participants with a clinical diagnosis of VM were included; in the CG, 13 randomly chosen healthy female individuals (26 ears) without auditory, vestibular, or migraine complaints were included.

### Procedure

Data for audiometry and vectoelectronystagmography of patients in the TG were obtained from medical records. The clinical data was obtained through the application of a protocol that questioned the participants regarding the following: symptoms and characterization of vertigo and migraine; symptoms associated with vertigo; auditory symptoms (tinnitus, auricular fullness, and hypoacusis).

Data on cVEMP and ECoG were obtained from records made in the ICS Chartr EP 200 (Otometrics) equipment. Examinations were performed in a silent environment, not acoustically treated. To obtain cVEMP data, we used a tone burst stimulus at a frequency of 500 Hz and intensity of 95 dB nHL, through insertion earphones.^1^, ^9^, ^10^ The capture of responses was obtained through surface electrodes fixed to the skin with electrolytic conduction paste, arranged in accordance with the British Society of Audiology recommendation (2014).^11^ The electrode impedance values were checked before recording, and the maximum allowed value was 5 KὨ.^1^, ^12^, ^13^

During the examination, participants remained lying on a stretcher with their heads turned contralaterally to the sound stimuli, maintaining tonic contraction of the sternocleidomastoid muscle using a cervical torsion monitor to standardize muscle contraction (50 μv to 70 μv). A total of 150 stimuli were administered at a rate of 5.1/sec with rarefied polarity. Responses were recorded separately in each ear in a 100 ms window with 5k gain, 10 Hz high pass filters, 1 kHz low pass filters, and Blackman envelope. The parameters considered were the presence of a biphasic wave with a Positive Peak (P1) followed by a Negative Peak (N1), latency of P1 and N1, and amplitude of interaural difference between these two peaks. An amplitude difference of up to 35% was considered symmetrical.^11^ To obtain data for ECoG, participants remained lying on a stretcher with surface electrodes arranged on the forehead (ground electrode), mastoid contralateral to the ear evaluated, and non-inverted electrode on the ear evaluated placed in the external auditory meatus in contact with the tympanic membrane. The click stimulus was used at an intensity of 90 dB nHL with 2,000 promediations at a rate of 7.1/s with gain of 50 K, high pass filter of 10 Hz, low pass filter of 1.5 kHz, and alternate polarity, recorded in a window of 10 ms. The parameter considered was the response to the acoustic stimulus composed of two mechanical-electric potentials of the cochlea: the Summation Potential (SP) and the compound Action Potential of the cochlear nerve (AP).^14^ The ratio of the amplitude of SP and AP (SP/AP) was considered normal when less than or equal to 35%.

### Statistical analysis

Descriptive data was expressed as mean, median, standard deviation, and minimum and maximum when appropriate. The Kolmogorov-Smirnov (KS) test was used to verify the normality of the data with the IBM-SPSS Statistics program (version 22.0, Inc., Chicago, IL). The Mann–Whitney test was used to compare measurements between the two groups. The Kruskal–Wallis test was used to compare measurements in more than two group. The Pearson Chi-Square test was used for analysis of categorical variables. Values of *p* < 0.05 were considered statistically significant.

## Results

The Test Group (TG) had 13 participants (26 ears), all of which were female with an average age of 44 ± 13 years. The control group had 13 healthy individuals (26 ears), all of which were female and did not have hearing, vestibular, or migraine complaints, with a mean age of 41 ± 11 years. There was no statistically significant difference in age between the respective groups.

All participants of the TG presented with vertigo and headache as clinical symptoms. For a majority (46.2%), vertigo lasted for minutes. Headache was predominantly unilateral (69.2%), pulsatile (84.6%), with a duration of days (69.2%) and described as accentuated in 69.2% of cases. These symptoms were accompanied by nausea (92.3%), photophobia (92.3%), phonophobia (69.2%), and auditory complaints (76.9%) (Table 1).

**Table 1:** Characterization of the clinical manifestations of the test group.

Clinical manifestations	N	%
Verification	13	100
Duration of dizziness		
**Minutes**	6	46.2
Hours	4	30,8
Days	3	23.1
Headache	13	100
Duration of headache		
**Minutes**	0	0
Hours	4	30.8
Days	9	69.2
Headache location		
**Unilateral**	9	69.2
Bilateral	4	30.8
Headache quality		
**Throbbing**	11	84.6
Non-throbbing	2	15.4
Intensity	4	30.8
Moderate	4	30.8
Accentuated	9	69.2
Impact on activities of daily living	9	69.2
Nausea	12	92.3
Vomiting	4	30,8
Photophobia	12	92.3
Phonophobia	9	69.2
Auditory complaints	10	76.9

N, Number; %, Percentage.

Among auditory complaints, tinnitus (61.53%) was the most frequent, followed by auricular fullness (23.07%) and hypoacusis (23.07%). Hearing loss was identified in 5 (38.46%) of the participants of the TG, with a total of 8 (30.76%) ears affected. The losses were sensorineural, mild, moderate, and severe.

Data from the vectoelectronystagmography examinations were available in the medical records of 10 participants of the TG (Table 2). The participants who did not have this information were invited to perform it but did not accept, claiming that the test was uncomfortable.

**Table 2:** Characterization of the vectoelectronystagmography examination.

Calibration		
Regular	10	100
Normal latency	8	80
Altered	2	20
Normal accuracy	8	80
Altered	2	20
Saccadic		
Regular	10	100
Normal latency	7	70
Altered	3	30
Normal accuracy	7	70
Altered	3	30
SN with missing EO	10	100
EC absent	7	70
EC present	3	30
SSN R absent	10	100
L absent	10	100
U absent	10	100
D absent	10	100
Pendular Screening I	9	90
II	1	10
Symmetrical ON	10	100
standard CT	3	30
LP > 19%.	2	20
DPN > 17%	1	10
Bilateral hyporeflexia	1	10
Not performed	3	30
Negative Dix – Hallpike	10	100

SN, Spontaneous Nystagmus; EO, Eyes Open; EC, Eyes Closed; SSN, Semi-Spontaneous Nystagmus; R, Right; L, Left; U, Up; D, Down; ON, Optokinetic Nystagmus; CT, Caloric Test; LP, Labyrinth Predominance; DPN, Directional Predominance of Nystagmus.

Regarding the performance of cVEMP and ECoG, participants of the TG were evaluated in the period between crises. For 41.66% (n = 5), the episodes occurred between 1 and 15 days prior to the examination; for 25% (n = 3), between 16 and 30 days; for 16.67% (n = 2), over 180 days previously; and for 8.33% (n = 1), between 31 and 180 days. Table 3 describes the values obtained for latency (P1 and N1) and amplitude (P1N1) of the cVEMP testing.

**Table 3:** P1 and N1 peak latencies and P1N1 amplitude in the test and control groups of the cVEMP tests.

Peaks	Mean	SD	Median	Minimum	Maximum
P1 (ms)					
CG	14.39	1.18	14.50	12.00	17.67
TG	15.19	1.49	13.00	13.00	19.67
N1 (ms)					
CG	21.45	2.17	20.50	18.00	25.83
TG	23.44	2.40	23.00	20.00	29.17
P1N1 (μv)					
CG	52.86	47.06	45.18	5.66	221.30
TG	91.59	61.66	82.72	6.00	217.35

cVEMP, Cervical Vestibular Evoked Myogenic Potential; TG, Test Group; CG, Control Group; SD, Standard Deviation.

**Fig. 1:**
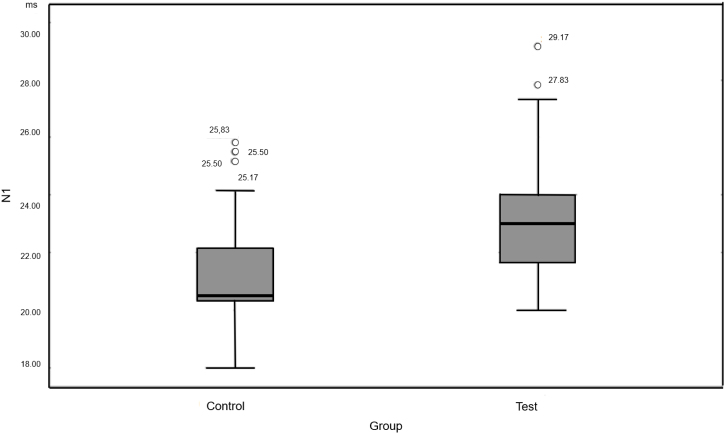
N1 latency. Source: Research data (2018). Mann–Whitney test Δ*p* = 0.002. Legend: ○, Outlier.

**Fig. 2:**
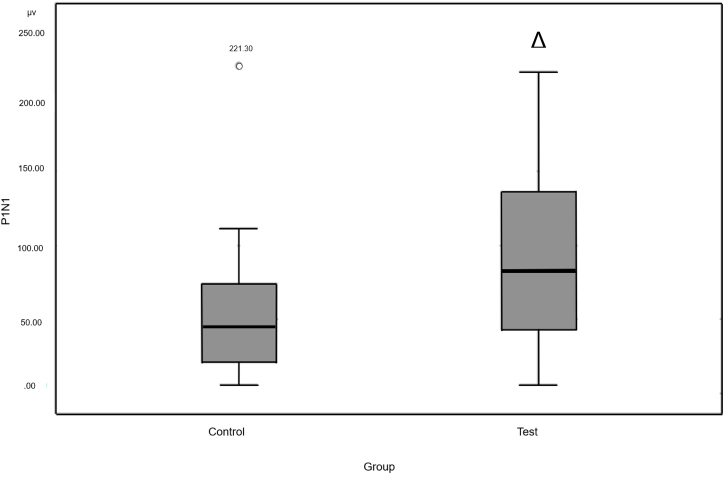
P1N1 amplitude. Source: Research data (2018). Mann–Whitney test Δ*p* = 0.017. Legend: ○, Outlier.

There was no statistically significant difference between TG and CG for P1 latency. For N1 latency, a statistically significant difference was observed (Fig. 1). Peak-to-peak amplitude P1N1 was quite varied in the groups. The mean amplitude in the TG was 91.59 μv, with a minimum of 6.00 μv and maximum of 217.31 μv. The mean CG was 52.86 μv, minimum was 5.66 μv, and maximum was 221.30 μv. The TG had a greater amplitude than that of the CG (Fig. 2).

**Fig. 3:**
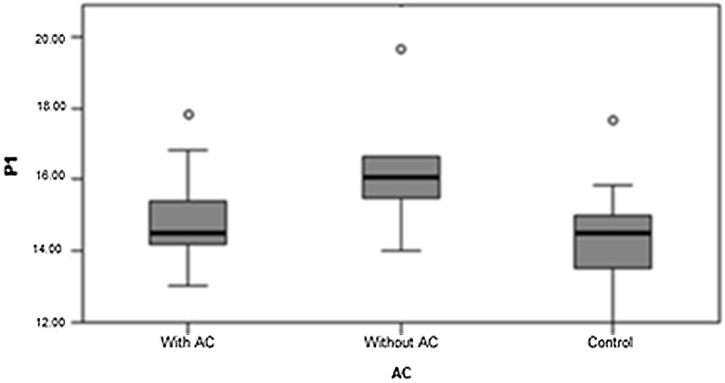
P1 latency in the TG with auditory complaint, TG without auditory complaint, and CG. Source: Research data. Kruskal–Wallis test Δ*p* = 0.021 (Without AC × Control). Legend: ○, Outlier; AC, Auditory Complaint.

**Fig. 4:**
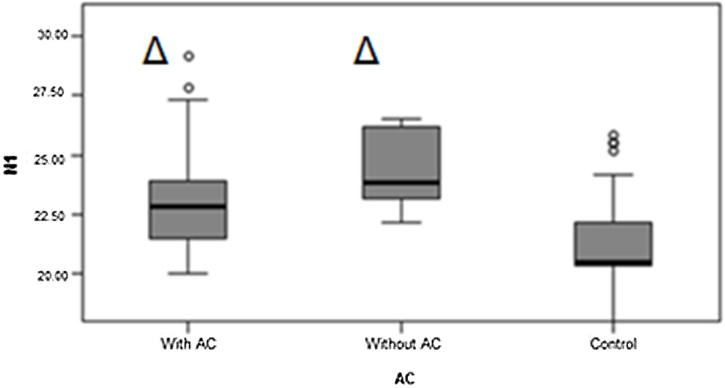
N1 latency in the TG with auditory complaint, TG without auditory complaint, and CG. Source: Research data. Kruskal–Wallis test Δ*p* = 0.013 (Without QA × Control); Δ*p* = 0.048 (With QA × Control). Legend: ○, Outlier; AC, Auditory Complaint.

**Fig. 5:**
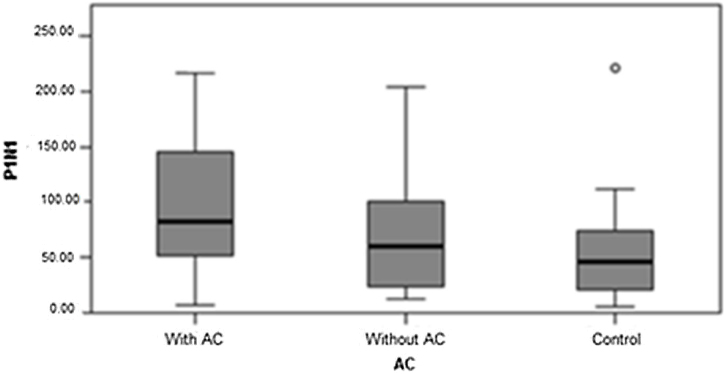
P1N1 amplitude in the TG with auditory complaint, TG without auditory complaint, and CG. Source: Research data. Kruskal–Wallis test Δ*p* = 0.032 (With QA × Control). Legend: ○, Outlier; AC, Auditory Complaint.

For further statistical analysis, the TG was subdivided into 10 participants with auditory complaints, for total of 20 ears, and three participants without auditory complaints, for a total of six ears. When comparing the three groups, significant differences were observed in the measurements of latency P1, N1, and amplitude P1N1 (Fig. 3, Fig. 4, Fig. 5). P1 latency was significantly higher in the TG without auditory complaints than in the TG with auditory complaints and CG (1.3).

N1 latency was higher in the TG without auditory complaint than in the CG, but it was not significantly different when compared to that in the TG with auditory complaint. Between the TG with auditory complaint and CG, N1 latency was higher in the TG with auditory complaint (Fig. 4). For the TG with auditory complaint, P1N1 amplitude was higher than that in the CG (Fig. 5).

Mean P1 latency for the TG was 14.85 ms ± 1.10 (RE) and 15.57 ± 1.80 ms (LE), respectively, when the ears were analyzed separately. Mean N1 latency was 22.90 ± 2.25 ms (OD) and 24.03 ± 2.52 ms (LE), respectively. There was no statistically significant difference in the values for the latencies of P1 and N1 of the RE (Right Ear), as well as in the amplitude P1N1 of the RE and LE (Left Ear) between groups. The latencies of P1 and N1 in the left ear were significantly higher in the TG (Table 4). Both the TG and CG presented three cases of P1N1 amplitude asymmetry (23.1%). The asymmetries were observed in two participants of the TG with auditory complaint, and in one participant of the TG without auditory complaint.

**Table 4:** cVEMP’s P1 and N1 peak latencies in right and left ear, and P1N1 amplitude in right and left ear in test and control groups.

cVEMP	TG					CG					
	Mean	Medium	SD	Min.	Max.	Mean	Medium	SD	Min.	Max.	p
P1 (ms)											
RE	14.85	14.50	1.10	13.00	16.67	14.67	14.83	1.38	12.00	17.67	0.738
LE	15.57	14.83	1.80	13.50	19.57	14.08	14.08	0.87	12.83	15.67	**0.014**
N1 (ms)											
RE	22.90	22.83	2.25	20.00	27.33	21.59	21.00	2.04	19.50	25.50	0.100
LE	24.03	23.67	2.52	21.00	29.17	21.31	20.50	2.37	18.00	25.83	**0.007**
P1N1 (μv)											
RE	96.57	88.82	70.67	6.00	217.35	43.11	45.14	30.63	5.66	107.70	0.054
LE	86.20	72.77	52.77	11.90	178.71	63.43	55.74	59.79	6.20	221.30	0.184
RE/LE	30.53	27.10	24.67	5.75	100.00	31.85	22.01	27.55	2.68	100.00	0.918

Mann–Whitney test.

cVEMP, Cervical Vestibular Evoked Myogenic Potential (cVEMP); RE, Right Ear; LE, Left Ear; TG, Test Group; CG, Control Group; SD, Standard Deviation; Min, Minimum; Max, Maximum.

ECoG revealed a mean of SP (0.18) and AP (0.75), with higher amplitudes in the TG, but no significant difference when compared to those in the CG (SP = 0.12; AP = 0.59).

There were no significant differences in the amplitude data of SP and AP for participants in the TG with auditory complaints (SP = 0.17; AP = 0.71), TG without auditory complaints (SP = 0.21; AP = 0.89), and CG (SP = 0.12; AP = 0.59).

When comparing ECoG testing results, it was evidenced that endolymphatic hydrops in the TG was double that in the CG, but this was not statistically significant. One participant in the TG had no response in the RE due to a degree of hearing loss, making it impossible to record the potential.

## Discussion

All participants were female, similarly to other studies.^15^, ^16^, ^17^, ^18^ All of them reported vertigo in at least one of their crises, most lasting minutes and not always concomitant with the headache. The participants reported a worsening of symptoms during the menstrual period. Previous studies have reported similar characteristics.^19^, ^20^, ^21^

Regarding auditory complaints, tinnitus was the most frequent, and hypoacusis and auricular fullness the least frequent. The presence of predominantly mild and sensorineural hearing loss has been reported in other studies.^21^, ^22^

Of the participants with hearing loss, two were over 60 years of age and one over 50 years of age, suggesting the possibility of associated presbycusis.^23^

Spontaneous nystagmus with eyes closed was a significant alteration, as well as labyrinthine hypofunction, which was present in 30% of those evaluated. There was no presence of nystagmus and/or dizziness in the Dix-Hallpike maneuver, contrary to the data reported in other studies.^22^, ^24^, ^25^

The variability of signs related to vectoelectronystagmography and physical evaluation is due to the fact that VM is a heterogeneous vestibular disorder that presents differences between periods of episodes and without episodes. All members of the TG were evaluated in the period between crises. It is likely that the absence of signs with central characteristics and positional nystagmus, which are reported in literature as more frequent,^21^, ^22^, ^24^, ^25^, ^26^ is because participants were evaluated outside the episode period.

The present study detected a significant increase in P1 latencies in the TG without auditory complaints in relation to the CG and in N1 latency in the TG without auditory complaints and in the TG with auditory complaints in relation to the CG, which may suggest the presence of central injury.^27^

When statistically analyzing the results of P1 and N1 latencies in the RE and LE, a significant increase was observed in the P1 and N1 latencies of the LE in the TG relative to that in the CG. Interaural difference in peak latency is associated with neuronal conduction speed. The asymmetry in this speed between sides, common in some neurological diseases, may explain this difference. Thus, disorders that interfere with neural conduction from the inner ear, brainstem, vestibulospinal tract, and second motor neurons may interfere with the response.^14^ Considering that the data revealed the presence of asymmetry in the cVEMP in 23.10% of those evaluated in the TG, it is possible that the participants had peripheral alterations, reinforcing previous findings.^23^ As there was no relationship between increased latency and cases of asymmetry, it may be assumed that participants presented different pathways of alterations (peripheral and central) to VM.

There was great variability in the values of P1N1 amplitude in TG and CG. The difference between the minimum and maximum values obtained in the P1N1 amplitude was associated with the age of the patients, where the lowest value of amplitude occurred in older participants and the highest value in younger participants, in both the test and control groups. There was a lack of response on one side in the older participants in the test and control group, aged 64 and 63 years, respectively. This absence can be justified due to ageing, which causes a decrease in strength and muscle mass. The TG presented increased P1N1 amplitude in relation to the CG, which may suggest the presence of hydrops at the saccular level.

The data obtained did not reveal a relationship between the changes identified by the cVEMP and ECoG. For many years, the presence of findings suggestive of endolymphatic hydrops were related to MD. The differential diagnosis of VM is MD. Thus, the test was used to evaluate the presence of signs of endolymphatic hydrops in the sample with VM, since the complaints among the diseases overlap. However, no significant changes in ECoG were observed. These results are similar to previous findings.^28^

The results, suggestive of endolymphatic hydrops to ECoG in the TG, was double the CG, but the difference was not statistically significant. All positive results were in the TG with auditory complaints. In participants who presented with bilateral absolute value hyporeflexia, the SP/AP ratio was altered bilaterally, suggesting a potential overlap of diseases.

The data from this study indicate that there was no association between changes in ECoG and hearing loss presented by five participants. The literature on ECoG in VM is sparse, and further research is needed to ensure that reliable data are obtained in examinations to aid in the diagnosis of VM and its differentiation from MD.

## Conclusions

VM and MD present themselves in similar ways, which may mean an association between disease pathophysiology. Heterogeneity of symptoms and findings can be observed among patients with VM, and, in the same patient, between one episode and another. This study showed the difficulty of finding specific markers for VM, which suggests that it is not a single disease, but is likely an association of different comorbidities or pathophysiologies present in patients. Vestibular migraine and endolymphatic hydrops can both be present in a patient, although ECoG data in our study was unable to find a relation between the two diseases. cVEMP found an increase in P1 and N1 latency that may suggest injury to the lower vestibular nerve or brainstem in VM. Further clinical investigation of VM symptoms remains necessary.

## Funding

This research did not receive any specific grant from funding agencies in the public, commercial, or not-for-profit sectors.

## Conflicts of interest

The authors declare no conflicts of interest.
